# Preparation of Hybrid Sol-Gel Materials Based on Living Cells of Microorganisms and Their Application in Nanotechnology

**DOI:** 10.3390/nano12071086

**Published:** 2022-03-25

**Authors:** Olga A. Kamanina, Evgeniya A. Saverina, Pavel V. Rybochkin, Vyacheslav A. Arlyapov, Anatoly N. Vereshchagin, Valentine P. Ananikov

**Affiliations:** 1 Tula State University, Lenin pr. 92, 300012 Tula, Russia; o.a.kamanina@gmail.com (O.A.K.); esaverina94@gmail.com (E.A.S.); rybochkin.pavel.vl@mail.ru (P.V.R.); v.a.arlyapov@gmail.com (V.A.A.); 2 N. D. Zelinsky Institute of Organic Chemistry, Leninsky pr. 47, 119991 Moscow, Russia

**Keywords:** nanotechnologies, sol-gel, biohybrid, yeast, bacteria, immobilization

## Abstract

Microorganism-cell-based biohybrid materials have attracted considerable attention over the last several decades. They are applied in a broad spectrum of areas, such as nanotechnologies, environmental biotechnology, biomedicine, synthetic chemistry, and bioelectronics. Sol-gel technology allows us to obtain a wide range of high-purity materials from nanopowders to thin-film coatings with high efficiency and low cost, which makes it one of the preferred techniques for creating organic-inorganic matrices for biocomponent immobilization. This review focuses on the synthesis and application of hybrid sol-gel materials obtained by encapsulation of microorganism cells in an inorganic matrix based on silicon, aluminum, and transition metals. The type of immobilized cells, precursors used, types of nanomaterials obtained, and their practical applications were analyzed in detail. In addition, techniques for increasing the microorganism effective time of functioning and the possibility of using sol-gel hybrid materials in catalysis are discussed.

## 1. Introduction

Over the last few years, the field of creating new hybrid materials has attracted great attention [1,2,3,4,5,6]. Of particular focus are biohybrid materials based on microbial cells. Within this general direction, material scientists study microorganism adaptation strategies to environmental changes. Due to these strategies, microbes can survive even under extremely tough conditions. Biomaterial encapsulation hinders the rapid removal of microorganisms and often their inactivation. Inspired by the versatility and strength of such biomaterials, scientists have developed hybrid materials for application in various areas, from agriculture and (environmental) biotechnology [7], biomedicine, and electrical engineering [8] to food production, synthetic chemistry, and bioelectronics [9,10].

**Figure 1:**
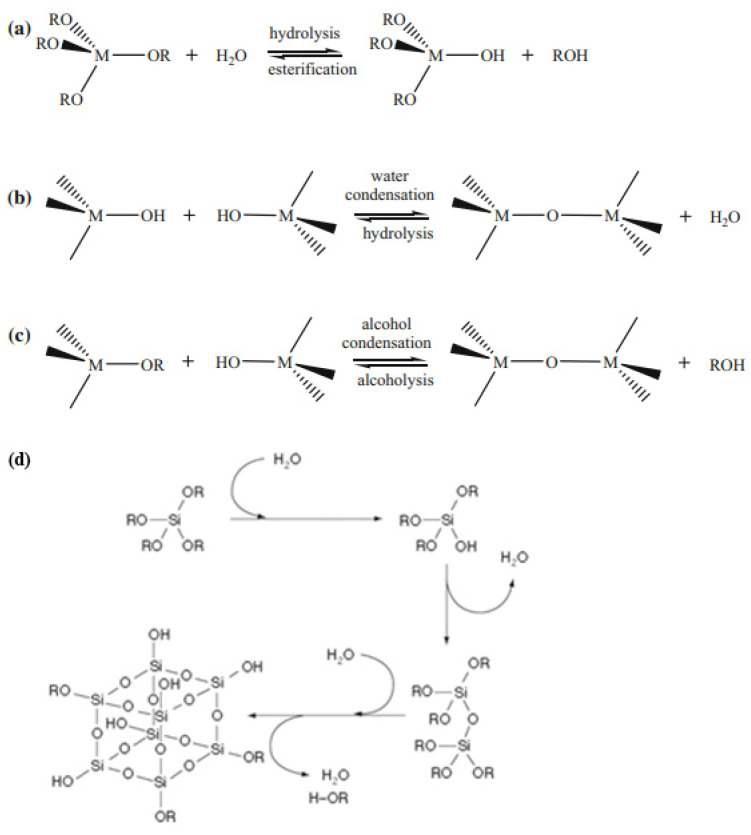
Hydrolysis (**a**) and condensation (**b**,**c**) of metal alkoxides. Reprinted with permission from [14]. (**d**) Formation of silica from tetra alkoxysilanes by hydrolysis and condensation. R alkyl, usually methyl, ethyl, or isopropyl groups. Reprinted with permission from [15].

Various approaches and methods are used to create hybrid materials, one of which is the sol-gel process, which allows porous materials to be obtained by converting sol to gel. The most common method of sol-gel synthesis is based on the controlled hydrolysis of alkoxides of silicon, aluminum, and transition metal M(OR)x (such as titanium, zirconium, tungsten, zinc, etc. (Figure 1)) and further polycondensation with the formation of oxoalkoxide derivatives, as described in detail [11].

The stage of condensed form generation during the hydrolysis of precursors determines the structure and morphology of the final products and is extremely important when forming sol-gel materials with desired characteristics. The structure of the forming sol-gel matrices depends on a large number of different factors, such as the presence or absence of substances with nonhydrolyzable MC bonds in the precursors, their concentration and ratio, the pH of the medium, acidic or basic catalyst, the presence of organic components, water-soluble polymers, and microorganism cells in the system [12,13].

**Figure 2:**
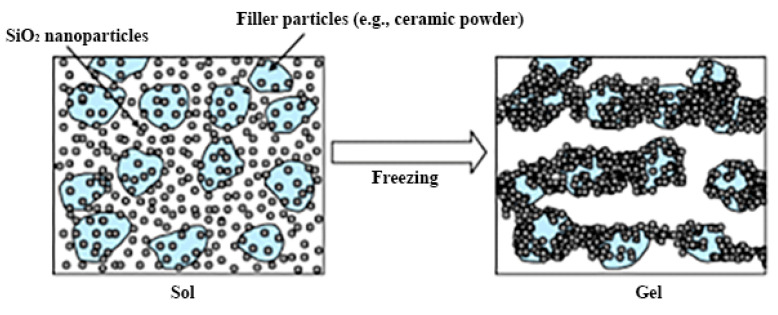
A schematic representation of the colloidal SiO_2_ nanosol dispersion irreversible transformation by the sol–gel transition caused by freezing. Reprinted with permission from [21].

The microstructure of the material produced by the sol-gel process depends on hydrolysis and condensation reactions, which are generally controlled by the pH of the solution. When using acid catalysis, hydrolysis proceeds faster than condensation, which usually begins when hydrolysis is completed [16,17]. Under basic catalysis conditions, condensation proceeds faster than hydrolysis, which leads to the formation of highly condensed species [18,19]. The hydrolysis rate of silicon alkoxides is minimal at pH = 7 and increases exponentially both at lower and higher pH values. This contrasts with the condensation rate, which is minimal at pH = 2 and peaks at approximately pH = 7 [20]. By varying the catalyst, it becomes possible to repeatedly influence the gelation time, porosity, density, and volumetric shrinkage during the drying process. The rate of sol-gel processes and the environmental pH directly influence the efficiency of biomaterial immobilization and its catalytic activity after immobilization. The formation of sol-gel matrices around microorganisms is also possible with irreversible transformation of the dispersion of colloidal SiO_2_ nanosols as a result of the sol-gel transition during freezing (Figure 2). It is important to take this into account when the materials are developed with desired properties. The use of microorganisms in combination with structures synthesized by the sol-gel technique makes it possible to use hybrid materials in medicine, ecology, materials science, and biotechnology.

In the last thirty years, there has been a gradual increase in the complexity of sol-gel processes for the immobilization of microorganisms of various taxonomic groups. This makes it possible to develop new application areas for sol-gel materials obtained by encapsulating microorganisms. In this review, we provide a brief description of the synthesis of such materials; a detailed description of such mechanisms can be found in the materials of articles [22,23,24].

## 2. The Classification of Hybrid Materials According to the Type of Immobilized Cells

### 2.1. Material Formation Procedure Optimization

For various fields of chemistry, biotechnology, or medicine, it is most advantageous to utilize living cells immobilized in a stable matrix as biocatalysts. This ensures the effective use of their physiological characteristics for obtaining secondary metabolites or in biotransformation. For industrial application and design of sensors based on whole cells, it is necessary to create their high density in a sufficiently small volume of matter, which can be achieved by encapsulating/incorporating cells into polymer matrices. Polymers containing both inorganic elements and organic components play an important role in the development of encapsulation techniques. Immobilization in such matrices makes it possible to achieve the highest efficiency biocatalysts, which is promising for their practical application in biotechnology.

As a result of the encapsulation process, living microorganism cells are surrounded by the formed silica shells, and the “cell in shell” structure has formed. Because the cells are limited in space, their growth occurs; therefore, the characteristics of the hybrid materials do not change [25,26].

As a result of a two-stage sol-gel process, alcohol toxic to cells is released during hydrolysis. Mild synthesis conditions under which alcohol was removed from the system in the first stage under vacuum on a rotary evaporator [27] or with a gas flow were developed to reduce toxicity and increase biocompatibility. The toxic effects of alcohol can also be eliminated using aqueous precursors such as sodium silicate and colloidal silicon dioxide [28].

**Figure 3:**
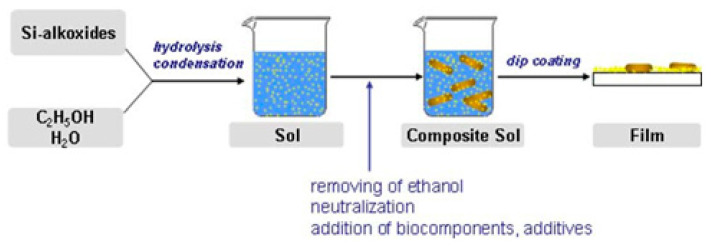
Preparation of silica layers with embedded microorganisms. Reprinted with permission from [29].

Reducing the toxic effects of both acids and alcohol on cells can be achieved using freeze drying. This process consists of freezing the cell suspension with ceramic powder and subsequent lyophilization. The addition of nutrients and cryoprotectants to the system while running the process at the optimum cooling rate improves cell viability. For example, the survival of *Rhodococcus ruber* was increased from 0.9% to 6.1% by the addition of trehalose solution [29] (Figure 3).

The traditional sol-gel process can be improved by introducing additives such as glycerol, glucose, and other sugars, or natural or synthetic polymers into the system to increase cell survival. They tend to increase the long-term stability of cells, as shown in the case of glycerol [30] and glucose [31]. Simultaneously, the immobilization process remains the same. These additives reduce the transparency of the matrix, which is important in the development of optical sensors.

With the exception of silica-based gels, microorganisms were encapsulated in oxide matrices of alumina, magnetite, titanium oxide, and zirconium [32,33,34,35,36]. Aqueous titanium and zirconium gels and their use for the encapsulation of microorganisms have been described [31,33,37,38,39]. Sols based on metal alkoxide were stabilized by the self-assembly of hydrophilic ligands, which ensured the formation of colorless, transparent aqueous sols. Encapsulated microorganisms were coated with a hydrated oxide and additionally included in the pores of the gel.

### 2.2. Immobilization of Bacteria by the Sol-Gel Method

The immobilization of bacteria in a sol-gel matrix leads to stabilization of the catalytic activity and makes it possible to repeatedly or continuously use the biocatalyst. The integration of microorganisms into sol-gel structures removes many limitations that arise during the working with free cell systems [21,40,41,42].

Over the last two decades, immobilization in a sol-gel matrix of microorganisms such as *Escherichia coli* [19,27,32,41], *Pseudomonas* [43,44,45], *Streptococcus* [46], and *Bacillus* [47] has been intensively studied (Figure 6). During the formation of sol-gel materials, the release of lower alcohols (ethanol or methanol) often occurs. This is the main cause of mass mortality of bacterial cells, in contrast to yeast, which is less affected by alcohols [27]. *Escherichia coli* bacteria have been efficiently encapsulated in organosilicon matrices [48]. Bacteria were isolated from each other in a layer of sol-gel material but still exhibited enzymatic activity against some substrates. However, the long-term stability of bacteria was 1 month, with a survival rate of approximately 10% even with the formation of sol-gel matrices under near physiological conditions at the required temperature, pH and ionic strength of the solution. To increase the viability of bacteria, various organic compounds are added during matrix formation, such as polyvinyl alcohol, gelatin, and glycerol [49]. It was shown that glycerol allowed the maintenance of the metabolic activity of almost 50% of bacteria after 1 month.

Kim et al. immobilized *Escherichia coli* bacteria in a silica sol-gel matrix and demonstrated their biological activity retention [50]. The study of the obtained material structure was carried out in the presence of various organic components, which increased the long-term performance of the biomaterial. The immobilization of *Escherichia coli* bacteria is used to explore the stability during storage and long-term continuous processes [51], to study the formation of various structures and the functioning of enzyme preparations during their immobilization [13], to study the effect of the resulting alcohol distillation on increasing cell viability [27,52], and to assess the effect of stress factors on bacterial immobilization [32] (Figure 6).

Immobilization of microorganisms in sol-gel matrices can be considered an alternative for long-term storage of nodule bacteria of the genus *Rhyzobium* at room temperature [53]. Sodium silicate was used as a precursor by Diazs’ group. In a continuation of the study [49], glycerol was used as the organic component. The bacteria immobilized in the sol-gel matrix retained their viability and catalytic activity for up to 360 days of storage at room temperature. In addition, the silicon matrix has been shown to have the ability to protect bacteria from acid attack.

**Figure 4:**
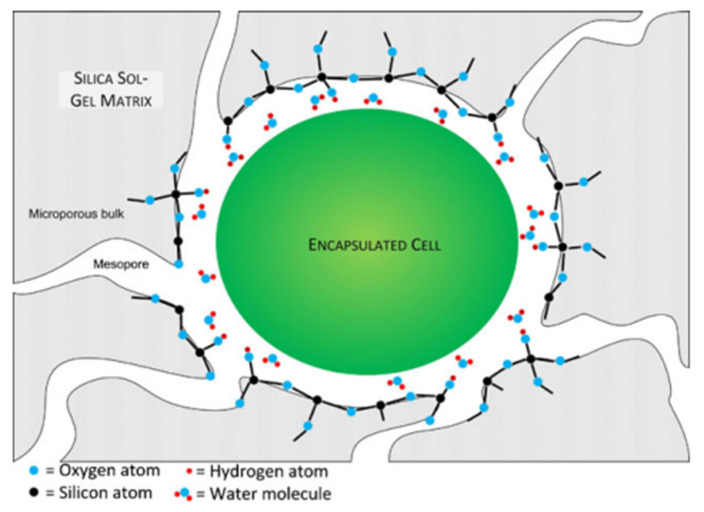
Schematic representation of a cyanobacterial cell encapsulated in silica gel (not to scale). The gel encloses the cell completely within a microporous bulk. The mesopores are large enough to allow diffusion of minerals and nutrients but small enough to contain the encapsulated cell. With alkoxide or aqueous precursors, the surface of the gel is likely composed of hydrophilic condensed silica with some uncondensed hydroxyl functional groups. Reprinted with permission from [26].

In view of their high abundance, cyanobacteria (Figure 4) are often used as model objects for studying various processes, including immobilization methods. In addition, they are important in biotechnology in the production of food additives, food, and pharmaceutical compounds and pigments, as well as in the production of biofuels and other products. The study of cyanobacteria encapsulation in a silicate matrix is described in [26].

Cells were immobilized in a sol-gel framework based on tetraethoxysilane (TEOS) under acid catalysis. Glycerol was used as an organic additive. As a result, cyanobacteria were encased in a porous organosilicon capsule, which, on the one hand, protects each cell from mechanical damage and, on the other hand, does not prevent the rapid diffusion of low molecular weight substances through the pores of the material [26].

**Figure 5:**
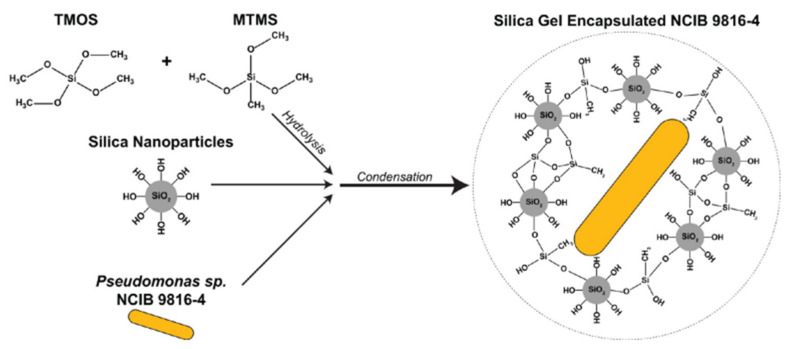
Synthesis process of the silica biogel through hydrolysis of TMOS and MTMS and condensation by mixing the hydrolyzed monomers with colloidal SNPs and *Pseudomonas* sp. NCIB 9816-4. Reprinted with permission from [45].

A silica-based adsorbent biogel was created by incorporating the bacteria *Pseudomonas* sp. NCIB 9816-4 that degrade a wide range of aromatic contaminants. The adsorbent matrix was synthesized using the silica precursors methyltrimethoxysilane (MTMS) and tetramethoxysilane (TMOS) (Figure 5).

**Figure 6:**
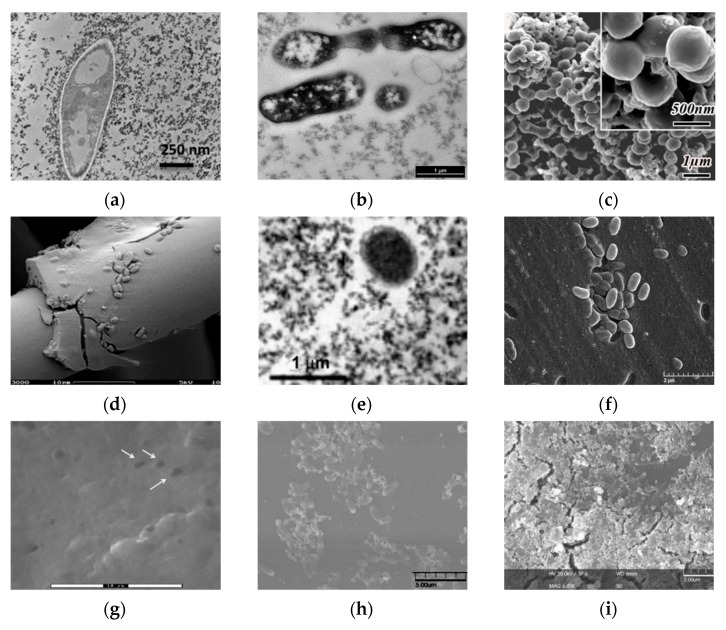

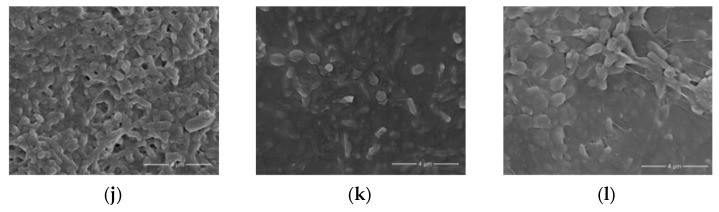
Micrographs of bacterial cells immobilized in various sol-gel matrices. (**a**) Transmission electron microscopy (TEM) image of *E. coli* bacteria entrapped in a ferrihydrite gel [30]; (**b**) transmission electron microscopy. Thin-cross-section TEM images of *E. coli* cells entrapped within an SS matrix after 24 h [32]; (**c**) field emission scanning electron microscopy images of bacteria/TiO_2_ gel hybrid spheres using *Str. Theromophilus* as templates, with the inset of a magnified image. The surface sol-gel deposition was repeated five times [54]; (**d**) different types of biocer-microstructure (scanning electron micrographs) carbon felt coated with a silica–*B. sphaericus* layer [55]; (**e**) transmission electron microscopy of the *E. coli* B 54,125 cell within an aqueous silica gel, SiO_2_–glycerol 10%, aged for one day [27]; (**f**) scanning electron microscopy (SEM) photos of mold silica gel-entrapped *P. aeruginosa* MR01 immediately after gel immobilization [43]; (**g**) SEM image of silica matrices with immobilized bacteria [44]; (**h**) SEM micrographs of *Sphingomonas* sp. cells [56]; (**i**) biohybrid of *Sphingomonas* sp.-(**f**) Si NP immobilized on microplate [56]; (**j**) SEM images of biofilm surface. Silica layer present after encapsulation. Representative electron microscopy images of *N. europaea* biofilm 30 min after encapsulation [57]; (**k**) 30 days after encapsulation [57]; (**l**) 90 days after encapsulation. Scale bars represent 4 mm [57]. Micrographs reprinted with permissions from the given references.

The encapsulated bacteria increase the rate of removal of the aromatic chemical mixture. Immobilized *Pseudomonas* bacteria have been successfully used to decolorize Remazol black, methylene orange, and benzyl orange, which are azo dyes commonly used in industrial processes [44] (Figure 6). The immobilized cells produced more than seven extracellular enzymes involved in the biodegradation of azo dyes. The reusability of immobilized bacteria has been evaluated through multiple experiments [44,45].

Immobilization of the methanotrophic bacterium *Methylomonas* sp. GYJ3 by the sol-gel technique enhances the activity of microorganisms at higher pH and temperature. However, the cells encaged by the sol-gel matrix based on MTMS had a lower activity compared to the activity of free cells [58]. At the same time, sol-gel matrices with immobilized Gram-positive *Rhodococcus ruber* bacteria demonstrated unchanged mechanical strength and good activity of immobilized cells when stored for several months at 4 °C [21]. The created ceramic composites can be reused for 12 months without loss of biological activity for bioremediation processes, as *Rhodococcus* spp. decompose a large number of pollutants that are difficult to oxidize, such as petroleum hydrocarbons, chlorinated, nitrogen-containing and other complex organic substances.

### 2.3. Immobilization of Yeast Cells by the Sol-Gel Method

The immobilization of yeast cells in a sol-gel matrix attracts much attention, as the alcohol released during the sol-gel reactions is not as harmful to yeast as to bacteria. In addition, yeast cells are often used as templates for the formation of porous inorganic structures [38].

The possibility of obtaining channel-like meso/macroporous TiO_2_, a potential anode material for lithium-ion batteries, has been described [31]. For this, a sol-gel process based on titanium tetraisopropoxide using yeast cells of *Saccharomyces cerevisiae* was utilized.

**Figure 7:**
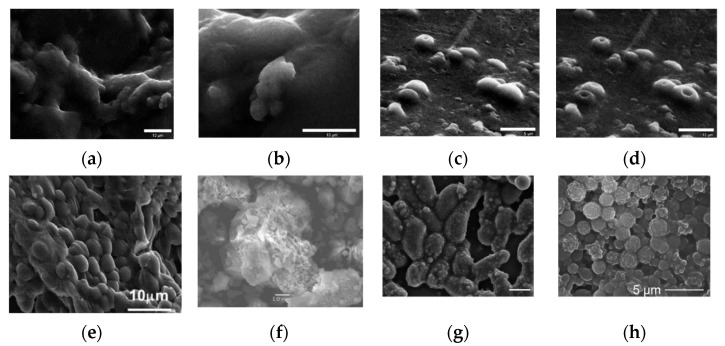
Micrographics of immobilized yeast cells in various sol-gel matrices. (**a**) SEM images of PGS-derived silica gels containing *S. cerevisiae* cells. (**b**) Typical long-range morphology with some shallowly encapsulated cells visible. Image (**d**) was collected several minutes after image (**c**), showing the development of depressions during imaging [34]. FE-SEM images of baker’s yeast encapsulated in sol–gel silica: (**e**) [63]. (**f**) Freshly harvested *Lodderomyceselongisporus* cells immobilized in a second generation (supported with hollow-silica microspheres) sol-gel system [67]. (**g**) *Cryptococcus curvatus* encapsulation in silica sol-gel. SEM micrographs showing the formation of 3D sol-gel biomatrix architecture when the ratio between the silane precursors (TEOS and MTES) (vol%) was 85:15. Scale bar, 5 µm [68]. (**h**) SEM micrograph showing the formation of a 3D structure hybrid material based on *Ogataea polymorpha* VKM Y-2559 cells encapsulated in an organosilica hydrogel MTES:TEOS 85:15 vol. % and PVA [25]. Micrographs reprinted with permissions from the given references.

The first work on the immobilization of the *Saccharomyces cerevisiae* whole cells in sol-gel was published in 1989 [59,60]. Since then, some *S. cerevisiae* cells have been used as models for studying yeast viability after encapsulation in a sol-gel matrix based on tetraethoxysilane, tetramethoxysilane, and diethoxymethylsilane [55,61,62,63] (Figure 7). Yeast *Saccharomyces cerevisiae* cells genetically engineered to produce yellow fluorescent protein in response to galactose were encapsulated in polyglycerol silicate matrices. The matrix consisted of glycerol, TEOS, and titanium isopropoxide [34]. A biohybrid of nanosilica and the model organism *Saccharomyces cerevisiae* was synthesized to remove mercury from an aqueous solution [64]. The efficiency of biosorption of heavy metals by microbial biomass is mainly related to the structure of the microorganism’s cell wall. Therefore, the structure and properties of the cell surface determine the nature of the interaction between the microorganism and the metal cation. The walls of yeast cells are negatively charged due to the presence of functional groups such as amino groups and phosphate and hydroxyl groups, which are involved in the binding of heavy metals. It is well known that among the various reactive compounds associated with cell walls, extracellular polymeric substances such as exopolysaccharides have a great ability to form complexes with heavy metals. The biohybrid has been shown to exhibit high Hg(II) adsorption capacity, demonstrating a rapid removal of more than 98 ± 2% of this contaminant in 30 min. The synthesized biohybrid material can be easily regenerated, and the efficiency of Hg(II) removal can be maintained when reused. In addition, the encapsulation of *Yarrowia lipolytica* in silicon matrices based on TEOS enables the development of a heterogeneous biomaterial that not only has the ability to remove Cr(III) and Cr(VI) pollutants from water without special pretreatment and with high efficiency but also to dispose of hydrocarbons in aqueous conditions. This process is possible due to *Yarrowia lipolytica’s* ability to produce various enzymes (proteases, lipases, and esterases), emulsifiers, and surfactants. The resulting biohybrid material has the advantages of a hydrophobic and porous structure and is able to achieve almost 100% removal efficiency of chromium and n-hexadecane ions in an aqueous medium [65].

It was found that in a hybrid material formed by silicon dioxide, polyvinyl alcohol, and 4-vinylpyridine with immobilized cells of the yeast *Trichosporon cutaneum*, a biocompatible microenvironment is formed, which contributes to the preservation of the viability of encapsulated cells [66]. Arthroconidia that have formed in the extracellular material play an important role in maintaining the long-term viability of microorganisms, which may be related to their ability to withstand environmental stresses. A biosensor based on the encapsulated yeast *Trichosporon cutaneum* was used to analyze the biochemical oxygen consumption in contaminated effluents.

Therefore, materials synthesis technology by the sol-gel method can be effectively used for the immobilization of a wide range of microorganisms, including Gram-positive and Gram-negative bacteria, as well as different types of yeast. At the same time, the sol-gel approach provides a biocompatible environment that protects cells from external influences, regardless of the microorganism type.

## 3. Classification of Hybrid Materials According to the Precursors Used in the Formation of the Sol-Gel Matrix

### 3.1. Silicon-Containing Precursors

Most often, alkoxides of the corresponding chemical elements are used as initial precursors for sol-gel reactions. In the case of silicon, tetramethoxysilane Si(OCH_3_)_4_ (TMOS) [45,69] and tetraethoxysilane Si(OCH_2_CH_3_)_4_ (TEOS) (Figure 1d) [27,34,65,67,70,71,72,73] are most commonly used.

It is often preferable to use substances that have a higher hydrolysis rate. This reaction rate of TMOS is much higher than that of TEOS. However, methanol is formed as a result of the reaction with TMOS, but the presence of this alcohol is not always allowed for the sol-gel process due to its toxicity [74]. Generally, the substitution pattern and therefore the organic residues of the precursors have a great influence on the kinetics of the sol-gel process. The utilization of TMOS or TEOS as precursors in a sol-gel synthesis at room temperature results in a 3D silica lattice. Sol-gel structures are often used in the formation of hybrid materials containing organic functional groups that are attached to an inorganic lattice [45]. For these purposes, various precursors are used, which include not only Si-OR groups that can be effectively hydrolyzed but also Si-C bonds that are stable to hydrolysis (Figure 1d).

**Figure 8:**
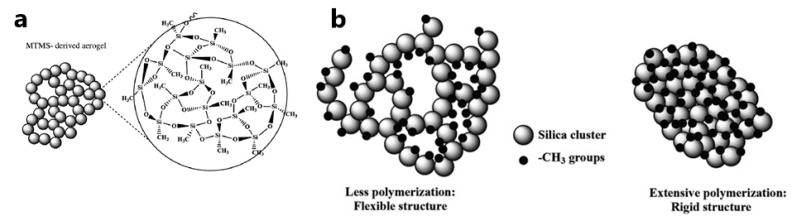
(**a**) Three-dimensional network of MTMS-derived aerogels with its detailed molecular structure. (**b**) Degree of polymerization of silanols exhibiting flexible structure and rigid structure. Reprinted with permission from [75].

As a result, the final material contains organic groups that have not participated in the sol-gel reactions. The use of this approach makes it easy to incorporate organic functional groups into the resulting organo-inorganic lattice. These functional groups can affect the chemical reactivity and polarity of the silica lattice and impart certain optical or electronic properties to the material. Branching and lengthening of the chain of the precursor substituent reduces the rate of hydrolysis [14,45]. However, it is necessary to use alkyl alkoxy silanes with nonhydrolyzable Si-C bonds as precursors to create more favorable conditions for the functioning of the biomaterial. The study of structures and the systematization of data about the obtained materials will make it possible in the future to predict the structure and properties of such matrices (Figure 8).

If the precursor used has at least three suitable crosslinking sites, then lattice formation is possible. The most commonly used organosilicon alkoxysilanes that have this ability are tetraalkoxysilane Si(OR)_4_ and trialkoxysilane (RO)_3_SiR′. Hydrolysis and condensations of alkoxides of the (RO)_2_SiR’_2_ or (RO)SiR’_3_ type lead to the formation of only chain molecules, where as mono-alkoxides form only dimers. The use of bis-alkoxysilane in combination with TEOS or TMOS can modify the resulting lattice with organic groups [13]. Mono-alkoxysilanes are rarely used in the formation of sol-gel materials; however, they can be useful for surface modification due to surface reactions [74]. Molecules that contain more than one silicon alkoxide group, such as systems containing two or more alkoxy groups (trialkoxide (R′O)_3_Si-R″H_2_-Si(OR‴)_3_), are also used in sol-gel processes [76]. Such precursors allow organic functional groups to be incorporated directly into the lattice of the material so that they become part of the lattice, where as molecules such as (RO)_3_SiR″ add the R″ functional group to the network.

Introducing both natural and synthetic polymers capable of forming spatial structures in aqueous media into the reaction mixture is often used in practice while modifying matrices. Polymers also serve as a seed to start gelation and to give flexibility to the final structure. In this regard, they can be considered structure directing agents (SDAs), which have a strong influence on the emerging structure. In the case when they are removed from the system (burning, etc.) after the completion of the synthetic process, i.e., in the case of template synthesis, they can also be considered templates.

Degradable synthetic or natural biopolymers, including PVA [25,66,77], poly(ε-caprolactone) (PCL) [78], gelatin [79], chitosan [80], and polyethylene glycol (PEG) [25,81], are widely used for biomedical purposes, as well as in biotechnological processes. PEG combined with sol-gel technology prevents excessive shrinkage of mesopores formed after the polymerization step, which can be harmful to cells [19]. Typically, these types of hybrid materials are prepared by mixing a polymer solution with a silica-based sol, followed by gel formation. In the case of obtaining a flexible structure of the sol-gel material, immobilized microorganisms are capable of division [32,34,82].

A high molar ratio of water to alkoxysilicate is often used [43,72] to negate the detrimental effect of ethanol on encapsulated cells (ethanol is a co-product of hydrolysis and condensation of alkoxide precursors). Therefore, a large amount of water leads to dilution of the alcohol solution, which ensures the biocompatibility of the process. Controlled evaporation of alcohol in vacuum is another way to eliminate its harmful effects [27]. Cells immobilized in silicagel are not only effective when reused [67] but also retain activity up to 90% [43], from 45 days [58] to 365 days [21,43].

The obtained sol-gel materials based on silicon with immobilized microorganisms were characterized using methods such as SEM, TEM, and nuclear magnetic resonance (NMR) [27]. The formation of a mesoporous material was shown, the position of cells in the material was found, and their encapsulation was proven [63].

### 3.2. Titanium-Containing Precursors

**Figure 9:**
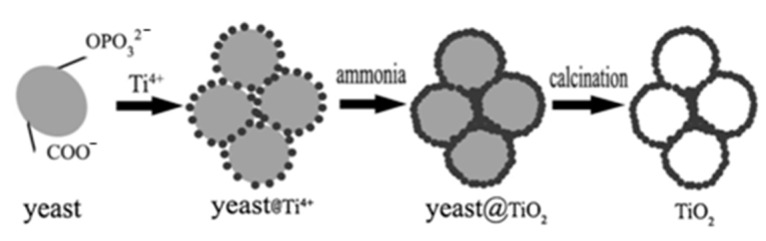
Schematic illustration of the formation of porous TiO_2_. Reprinted with permission from [38].

Titanium dioxide obtained by the sol-gel technique is rarely used for the immobilization of living microorganism cells due to its pronounced antimicrobial properties [83]. Most often, immobilization of microorganisms in such matrices is carried out to obtain a hierarchical mesoporous structure after annealing of microorganisms (Figure 9) [31,38].

However, the modification of titanium dioxide with silicon dioxide [35] allows the material to be used to remove arsenic from effluents. However, this may lead to a decrease in the viability of immobilized microorganisms [34].

Titanium dioxide obtained by the sol-gel method is most commonly used to modify capsules with immobilized cells to increase mechanical stability [84]. The hybrid alginate/TiO_2_ microcapsule showed improved mechanical stability compared to the pure alginate microcapsule, which makes it an ideal candidate for cell reservoirs. The formed microcapsules showed high biocompatibility with model human HepG2 cells [84], which are used as test materials in the creation of tissue engineering constructs. Such a microcapsule had a mesoporous structure, which is a key parameter allowing the diffusion of nutrients and metabolites. Subsequently, modification of the developed capsule with silicon dioxide made it possible to study the in vivo biocompatibility and stability of alginate/TiO_2_ hybrid microcapsules and immune isolation of captured HepG2 cells to assess their potential for cell therapy [85].

### 3.3. Aluminum-Containing Precursors

Methods for obtaining aluminum oxide hydrogels by the sol-gel technique are described. The resulting hydrogels provide long-term viability for encapsulated microorganisms [36,86]. Alumina gels were chosen because the chemical composition of the Al_2_O_3_ sol-gel has some similarities with the chemical composition of silica. In addition, it is well known that Al^3+^ ions are toxic to a large number of living systems, whereas Al_2_O_3_ is widely used in medicine. The formation of gels is very rapid, faster than using silica, from 2 min [36] to 15 min [86], which can cause significant stress for living cells. Therefore, for better adaptation and functioning of microorganisms, structure-directing agents such as glycerol are added [36].

### 3.4. Cerium-Containing Precursors

Nanomaterials based on cerium oxide (CeO_2_) are technologically important due to their valuable properties and are widely used in various fields from engineering to life sciences. At low temperatures, cerium oxides exhibit antimicrobial activity against some bacteria, destroying the cell walls of microorganisms [87]. At the same time, CeO_2_ particles are less biocompatible with a wide range of microorganisms. Moreover, their inherent redox activity can help eliminate reactive free radicals that stress living microbial cells.

Silica hydrogels containing CeO_2_ nanoparticles have pronounced protective properties. Immobilization of the model photosynthetic microalgae *Chlorella vulgaris* showed effective and long-term retention and growth of microorganisms. These properties have been evaluated under conditions of harmful ultraviolet radiation as well as in the presence of H_2_O_2_ [39]. Previously, CeO_2_ nanoparticles were shown to have good optical properties in terms of their ability to photoabsorb UV light with an efficiency of up to 89% [33], and there is no visible light scattering [39].

Various silicon derivatives, in particular its alkoxides, are most often used as precursors for sol-gel processes. The use of metal derivatives to obtain hybrid materials based on living cells of microorganisms is limited by their toxicity and antimicrobial properties. However, this problem can be partially solved through the use of biocompatible structure-controlling agents.

## 4. Classification of Hybrid Materials According to the Type of Nanomaterial Obtained

### 4.1. Bioceramics

A number of scientific studies describe the immobilization of living cells in ceramic composites with further possible use for the remediation of organic pollutants and heavy metal ions. It has been shown that bioceramics based on silicon precursors with immobilized cells of microorganisms can effectively remove phenols [21] and dyes due to a combination of adsorption and decomposition processes [71,88], as well as the biosorption of heavy metals [47,89]. The efficiency of biocatalysts obtained by immobilizing *Trametes versicolor* cells in ceramic sol-gel matrices using TEOS was evaluated using methylene blue and malachite green dyes as the model organic micropollutants. The results demonstrated that this bioceramic material is able to effectively remove dyes by a combination of adsorption and degradation processes.

Immobilization of the biomaterial in ceramic coatings based on aluminosilicates [21] or in silicagels based on silicon dioxide [71,90] allows not only preservation of the catalytic activity of the biocomponent but also acquisition of a strong and time-stable biocomposite structure. Immobilization of phenol-decomposing bacteria *Rhodococcus ruber* into aluminosilicates enables bioceramics with durability of more than half a year to be obtained [21]. The use of a combination of algae and silica when creating bioceramic coatings makes it possible to obtain a biomaterial with high mechanical stability, an algae content of 30–50%, and a total porosity of 40–60% [89].

The cells immobilized in bioceramic material can be used in biotechnological industries [31,68,91]. Yeast cells were immobilized in titanium tetraisopropoxide, resulting in porous bioceramic structures. Further processing of the precursor made it possible to obtain a channel-like meso/macroporous anode material that can be used for a lithium-ion battery [31]. Furthermore, bioceramic materials consisting of *Haematococcus pluvialis* microalgae living cells immobilized in silica sol-gel layers can be used for the biotechnological production of astaxanthin. Böttcher and colleagues demonstrated that the use of Fe^2+^ compounds in combination with NaCl or hydrogen peroxide as stress factors causes a strong increase in the formation of astaxanthin during cultivation [68].

### 4.2. Thin Films

**Figure 10:**
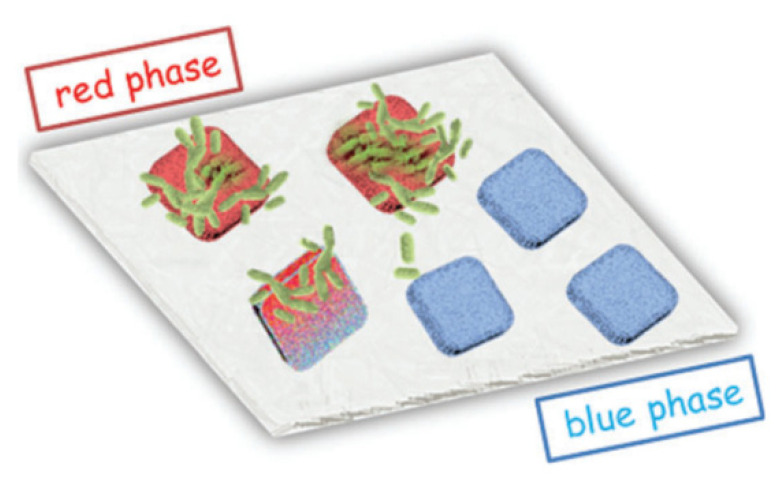
The red and blue chromatic solgel/polydiacetylene (PDA) thin films, assembled through a dip-coating technique, enable in situ colorimetric and fluorescent detection of bacterial biofilm formation (see figure). Interestingly, the gel-embedded PDA domains promote biofilm accumulation. The sol-gel/PDA assembly can also be employed for high-throughput screening of biofilm inhibitors. Reprinted with permission from [82].

Thin films are created by biomaterial immobilization in modified tetraethoxysilane. The use of various compounds as modifying additives to impart the necessary properties to the material is described. Thus, the use of polydiacetylene [92] as a modifier promotes the rapid growth of bacterial biofilms (Figure 10); cytochrome [93] can serve as an electronic mediator between bacteria and the electrode surface, where as chitosan and polyethylene glycol [94] can be used as modifiers to improve the functionality and, as a result, the catalytic activity of *Pseudomonas aeruginosa* BN10 cells.

Additionally, TEOS can be modified with glycerol [95], which acts as a protective agent for encapsulated cells and can serve as the sole source of carbon for *S. cerevisiae* under aerobic conditions, and with sodium alginate [96], which, in combination with silica, improves the stability of *Chlorella vulgaris* cells in saline solutions and allows us to demonstrate the stable reproducibility of the obtained materials. Thin films obtained by the sol-gel method can be used to form biofilms. Such sol-gel films represent a new universal platform for the advancement of bacterial biofilms and their in situ analysis. The *Pseudomonas aeruginosa* BN10 cell immobilization method was used to assess their efficiency in terms of biodegradation and the protective effect of microorganisms against large amounts of hydrocarbons [94]. The results obtained showed that the organic part in the synthesized hybrids is important for creating the microstructure and certain properties.

Thin films based on silica can be used as materials for biosensors [97,98]. Microalgae *Mesotaenium* sp. and cyanobacteria *Synechococcus* sp. cells were immobilized by the sol-gel technique using a thin layer of silicon dioxide [97]. Timur’s group [98] created and characterized a mediator whole-cell biosensor with acetic acid bacteria *Gluconobacter oxydans*. Microorganisms were immobilized on a graphite electrode using a hybrid composite based on TEOS obtained by the sol-gel technique.

A number of scientific studies are dedicated to studying the preservation of the viability of cells immobilized in thin layers of sol-gel matrices using various methods. Immobilization of the biomaterial in thin silica films results in an increase in the long-term stability of biofilms from 3 [99] to 8 [96,97] and 12 weeks [57], depending on the biocomposite. At the same time, in some cases [100], the apparent cell density increased almost 3-fold after 3 weeks, and cell viability slightly increased to 70 ± 10%. Etienne et al. have shown that the presence of chitosan, trehalose, and polyethylene glycol additives significantly improves the viability of *E. coli* cells in the electrodeposited matrix for 1 month after encapsulation. The bioluminescent activity of *E. coli* MG1655 pUCD607 was preserved in approximately 50% of the cells present in such composite films [99].

According to research [57,69,99], a thin membrane of silica or its modifications allows the diffusion of nutrients and cellular products, maintaining cell viability.

Therefore, bioceramics and thin films are the main types of hybrid materials obtained by the sol-gel method. Bioceramics obtained using silicon precursors and immobilized cells of microorganisms can be effectively used for the sorption of organic dyes, phenolic compounds, and heavy metals. Thin films based on silicon precursors can be used as a matrix for the formation of biofilms and as a material for creating sensors for electrochemical and optical biosensors.

## 5. Classification of Hybrid Materials According to the Application of the Resulting Nanomaterial

### 5.1. In Ecology

**Figure 11:**
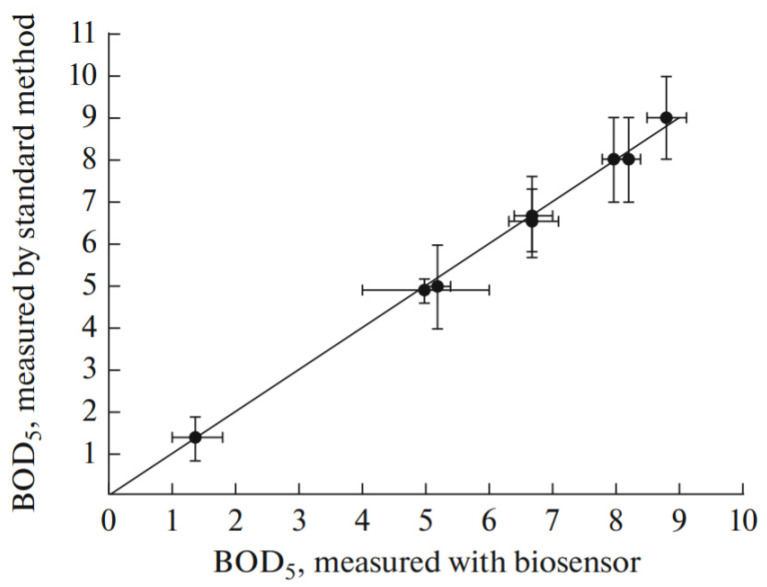
Correlation of biosensor BOD measurements with those carried out by the standard method. Reprinted with permission from [102].

The content of bioavailable pollutants in aquatic systems is an important criterion in the evaluation of the toxic effects of compounds accumulated in the environment. Hydrogel-immobilized microorganisms have a higher tolerance to toxic contaminants due to their protective capsule. However, they suffer from a low transport rate of substances that contribute to efficient cell functioning (oxygen and nutrients). This occurs due to an additional mass transfer barrier [41]. Immobilized microorganisms are used for toxic pollutant bioremediation. The contaminant can be partially removed by passive adsorption on the matrix material and partially by catalytic reactions of microorganisms. These mechanisms can work simultaneously. Currently, various studies have been carried out to create sensors for determining the concentrations of BOD and heavy metals. The use of such systems is very important in biomotoring because a part of the sol-gel matrix with immobilized microorganisms has protective properties [91,95,97,101,102] (Figure 11).

The authors of several studies [66,103,104,105] developed sensors for the rapid determination of BOD. Microorganisms were immobilized using matrices based on silicon dioxide modified with the mediator ferrocene [103], polyvinyl alcohol [104], and a copolymer of polyvinyl alcohol with 4-vinylpyridine [66,105]. The range of biodegradable substrates can be extended by including coimmobilized microorganisms. The following mixtures of microorganisms were used to expand the substrate specificity profile: *E. marius*, *B. horikoshii*, and *H. Marina* [103]; three different species of sea water microorganisms [104]; *Trichosporon cutaneum;* and *Bacillus subtilis* [105]. In [66], an organic-inorganic hybrid material, which consists of silicon dioxide and a copolymer of polyvinyl alcohol and 4-vinylpyridine, was used to immobilize cells of the *Trichosporon cutaneum* strain. It was found that a biocompatible microenvironment formed in the biomatrix contributes to the long-term viability of the captured cells. The mechanism of immobilized cell long-term viability was studied using confocal laser scanning microscopy. It was shown that Arthroconidia formed in extracellular material are essential for maintaining the long-term viability of microorganisms, which is probably caused by Arthroconidia’s ability to resist environmental stresses [66]. The resulting biosensors demonstrated high reproducibility and long-term stability. The results were simultaneously compared with the traditional BOD 5 measuring method and other sensory methods for measuring BOD. The determination results obtained for natural sea water correlate with those obtained from conventional BOD 5 analysis. Thus, it was shown that the developed biosensors are suitable for determining BOD.

**Figure 12:**
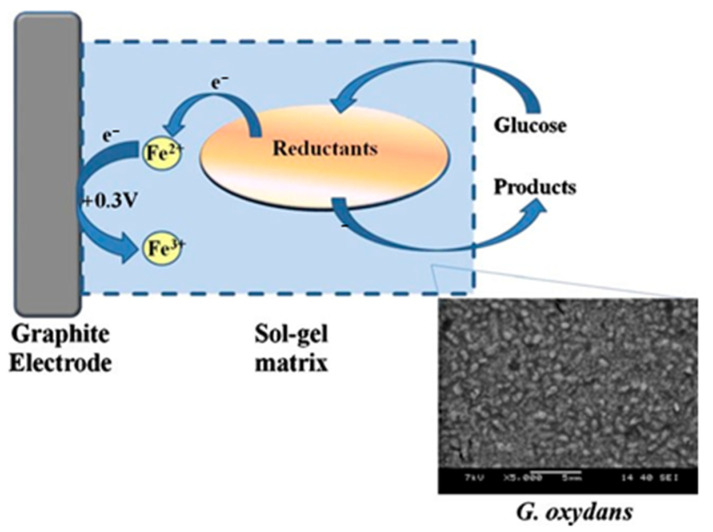
Schematic illustration of the principal microbial biosensor including SEM images *of G. oxydans* (with ×5000 magnification). Reprinted with permission from [98].

Timur et al. developed a mediated whole-cell biosensor based on *Gluconobacter oxydans* cells [98]. *G.oxydans* are Gram-negative bacteria that are actively used in sensory systems to detect polyols, sugars, and alcohols [106]. Bacterial cells were immobilized on the surface of graphite electrodes via a sol-gel (tetraethyl orthosilicate)/chitosan hybrid composite modified with gold nanoparticles (Figure 12). The resulting biosensor for the determination of ethanol and glucose demonstrated advantages such as a fast amperometric response, high sensitivity, and good repeatability. In addition, the authors suggest that the obtained material can be used in biofuel cell applications as a microbial cathode.

Microorganisms immobilized in silicate matrices and hydrogels are able to utilize heavy metals such as cadmium [97], mercury [64], chromium [97], zinc [97], and other pollutants, thereby purifying the environment. As an example, an optical biosensor [97] using two strains of microalgae *Mesotaenium* sp. and a strain of cyanobacteria *Synechococcus* sp. to detect Cd^2+^, Cr^6+^ and Zn^2+^ in aqueous systems. In addition, a whole-cell biosensor for the detection of bioavailable heavy metals in soils was created. This sensor contains a bacterial strain of *Bacillus megaterium* VR1 immobilized in a silica gel matrix. This strain is sensitive to several heavy metals [47]. Microalgae-cyanobacteria immobilized in silicon dioxide can be used for environmental monitoring of water samples from industrial effluents discharged into inland waters. They can be used to detect the bioavailable fraction of heavy metals. The mesoporous silica matrix reinforced with nanomullite was effectively used for biomaterial immobilization to preserve the long-term viability and enzymatic activity of the biocomponent. In this case, arsenic was efficiently removed by the bacterium *Ralstonia eutropha* MTCC 2487 [90], which remained viable in the obtained matrix for up to 120 days. Such biosensors and biofilters have great potential for monitoring heavy metal toxicity.

The development of long-term storage biofilters is significant for the treatment of contaminated water. These filters contain microorganisms that can degrade compounds that are difficult to oxidize, such as fuel oxygenates methyl tert-butyl ether (MTBE) and ethyl tert-butyl ether (ETBE) [71], n-hexadecane and chromium ions [65], and methyl parathion [56].

The integration of microorganisms into matrices obtained by the sol-gel method makes it possible to increase the storage time and functioning of biohybrids. For example, an optical sensor was created by functionalization of silica nanoparticles with polyethyleneimine and immobilization of *Sphingomonas* sp. This sensor was used to detect methyl parathion [56]. In this case, the storage stability of the biohybrid was enhanced from 18 to 180 days. In addition, *Aquincolatertiaricarbonis* L108 cells, capable of biodegrading fuel oxygenates of methyl tert-butyl ether and ethyl tert-butyl ether immobilized in a sol-gel coating on porous silica granules, can be stored in a humid atmosphere for 8 months without a significant decrease in their metabolic activity [71].

Mesoporous silica nanoparticles have an adsorption capacity, albeit limited, for heavy metal ions due to their characteristics. Thus, biohybrid material was obtained by encapsulating *Yarrowia lipolytica* in silicon matrices [65]. The resulting material was able to remove Cr(III) and Cr(VI) pollutants from water with high efficiency and without special pretreatment, which makes it convenient for practical use. The initial adsorption capacity of *Y. lipolytica* for Cr(III) and Cr(VI) is enhanced by the introduction of silicon dioxide. The yeast *Y. lipolytica* was tested for n-alkane removal efficiency in water with n-hexadecane as a typical contaminant. The improved n-hexadecane removal capability is due to the high surface area of the hybrid materials as well as the hydrophobic surface interaction between biosilica and n-hexadecane, which enhances the adsorption of the latter by the biosilica-yeast hybrid material. With the advantages of hydrophobicity and porous structure, this hybrid material exhibits enhanced handling capabilities for chromium and n-hexadecane ions, reaching nearly 100% removal efficiency for both contaminants.

Another application of sol-gel materials containing living bacterial cells of *Pseudomonas* sp. is their use for decolorizing water containing azo dyes. It was observed that immobilized bacteria produced more than seven times more extracellular enzymes involved in the biodegradation of azo dyes. The reusability of the material was evaluated through repeated decolorization experiments. The decolorization degree was over 75%, 79%, and 83% for Remazol black, methyl orange, and benzyl orange, respectively. Immobilized bacteria have the advantages of high viable cell density, high stability, and increased reaction time. Thus, the biocomposite can be used as an economical and effective agent for effluent dye cleaning [44]. In addition, *Pseudomonas* bacterial cells immobilized in sol-gel matrices using TEOS can be utilized for the production of biosurfactants, as described in [43]. The viability of immobilized cells was maintained at ≥84% for 365 days after immobilization.

Immobilized microorganisms are able to effectively dispose of organic compounds such as 4-phenylbutan-2-amine or heptan-2-amine [107] in a continuous flow mode. At the same time, selective reduction of prochiral ketones and acyloin condensation of benzaldehyde with yields from moderate 20% to great 99% were observed during the joint immobilization of yeast cells of *Lodderomyceselongisporus*, *Pichia carsonii*, *Candida norvegica*, and *Debaryomyces fabryi* in a sol-gel matrix [108].

Sometimes, to remove pollutants in one medium, enzymes are used in combination with materials obtained by the sol-gel method [109,110]. In previous work [109], magnetic nanoparticles modified with siloxane layers and having functional groups (amino groups and thiol groups) immobilized the urease enzyme. The activity of the immobilized enzyme during urea hydrolysis reached levels characteristic of the native enzyme, and its long-term stability allows its repeated use in the analysis and detoxification of biofluids.

### 5.2. In Medicine

Silica-based sol-gel materials have many properties of an ideal material for tissue regeneration, such as high surface area and porous structure in terms of overall porosity and pore size, which promote cell-material interactions and cell invasion. Studies of these materials have shown that the surface area is increased due to the porous structure, which provides a higher rate of tissue binding.

**Figure 13:**
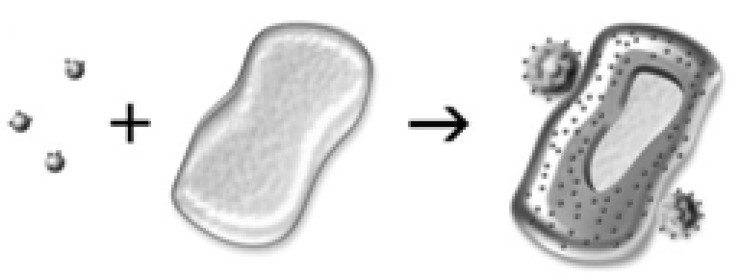
The mechanism of encapsulation of a cell in a metal oxide gel; the coalescence of MTSALs around a living cell with formation of a continuous oxide shell. Reprinted with permission from [37].

Bioceramics based on sol-gel materials have great potential for use as coatings on metal substrates to provide a high degree of biocompatibility and promote rapid recovery with minimal biological side effects. However, compatibility is only one aspect of biomedical applications based on sol-gel methods. Undoubtedly, one of the main advantages of using sol-gel approaches to the production of bioactive coatings is the absence of the necessity to maintain high temperatures during the synthetic process. Relatively low synthesis temperatures avoid the complications of applying bioactive coatings, such as mismatched thermal expansion coefficients found in conventional coatings, which can lead to cracking and poor interphase interaction. Matrices based on titanium dioxide or TEOS have very low cytotoxicity with respect to the cell lines used compared to other materials [111] (Figure 13). Thus, the sol-gel method is simple, stable, cost-effective, and scalable to facilitate future industrial production and clinical translocation [112].

With versatile and customizable structures, mesoporous materials based on silica [70,85], titanium [84], or zinc [113] are capable of loading various molecules, including pharmaceuticals, therapeutic peptides, proteins, and genes. Mesoporous silicate nanomaterials have been used as a drug delivery system for various kinds of medications with different hydrophobic or hydrophilic properties, molecular weights, and biomedical effects [114].

**Figure 14:**
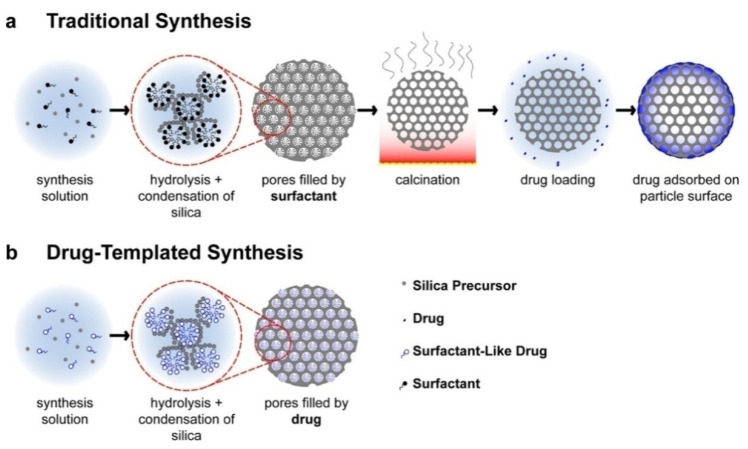
Traditional concentration-gradient-driven drug loading (**a**) and drug-templated synthesis of MSNs (**b**). Reprinted with permission from [115].

These medications include commonly used agents such as ibuprofen, doxorubicin, camptothecin, cisplatin, and alendronate. Peptide and protein preparations have been developed as effective therapeutic agents for many medical applications [84], including cancer therapy [113], vaccination and regenerative medicine [85]. Mesoporous silicates can protect biomolecules from premature degradation due to their porous structure. However, proteins are difficult to deliver, in part because of their inherently high molecular weight and fragile structure that must be maintained to retain activity [11]. Nevertheless, interest in delivery systems based on silicon dioxide or titanium dioxide [84,85] for the oral delivery of drugs, biomolecules, or cells is continuously growing. Active substance carriers can be synthesized in two ways: by encapsulating the biomaterial in presynthesized silica or by encapsulating and forming silica in one step (Figure 14). Silica production by sol-gel technology is carried out at a relatively low temperature (<40 °C), which makes the process compatible with the manipulation of thermosensitive drugs, peptides, proteins, and, in particular, cells. A simple modification of the silica surface allows controlled release of contents when exposed to changes in pH conditions and/or the presence of enzymes.

The highly porous structure of sol-gel silica makes it an ideal candidate as a matrix for a targeted drug delivery system designed to achieve gastric retention. Mesoporous silica nanoparticles mixed with sodium bicarbonate, as a gas generating agent, and a cellulose-derived polymer were used to prepare tablets for curcumin and captopril, hydrophobic and hydrophilic model drugs, respectively. The resulting tablets can be kept in the stomach for up to 12 h. Highly porous calcium silicate and aluminosilicate have also been successfully used in the manufacture of a drug delivery system. Thus, they have been used to include repaglinide, a hypoglycemic agent with poor absorption in the upper intestinal tract, and methotrexate, an anticancer agent with a short half-life of 2 h [112].

The main function of the biocompatible encapsulating material is to provide a barrier between the body’s immune system and the implant, as well as to provide the structure with the necessary mechanical stability. The simplest implant design is a small bubble of functioning tissue.

Isolated mouse islet cells were encapsulated in silica shells. The resulting islet capsules were implanted intraperitoneally into mice with diabetes induced by streptozotocin injections. At least one mouse maintained full blood glucose control for 10 weeks before the activity of the implant was lost. This work is preliminary, and no statistical results are available. This study is important primarily as an innovative application of sol-gel silicate chemistry in the field of organ transplantation.

One of the most promising and widely presented technologies for obtaining silica-encapsulated cells is the BioSil method developed by Giovanna Carturan. According to the BioSil technology, the sol-gel precursors are transported to the place of encapsulation in the gas phase. The starting reagents are usually a mixture of tetramethoxysilane and methyltrimethoxysilane. This strategy allows precise control of the thickness, porosity, pore size, and composition of the resulting siliceous membrane. Hydrolysis of precursors occurs in the surface water layer covering cells or cell clusters. The gas stream removes harmful hydrolysis products, leaving a smooth, fairly flexible silicate membrane. It has been reported that isolated rat pancreatic islets can be safely covered with a silicate membrane without loss of viability and function. Nevertheless, the authors noted some damage to cells in the peripheral regions of the islet, as well as a decrease in insulin release by approximately two times compared with uncoated tissue. Islands encapsulated in silica were surgically transplanted under the left kidney capsule in incompatible rats. This operation provided the diabetic recipient with adequate glycemic control for at least 8 weeks until the experiment was terminated. Unencapsulated islets collapsed within the first week [15].

It can be concluded that the developed silica, titanium, or zinc particles are biologically inert and biocompatible, making them suitable for biomedical uses, including drug delivery and release applications.

### 5.3. For Batteries

**Figure 15:**
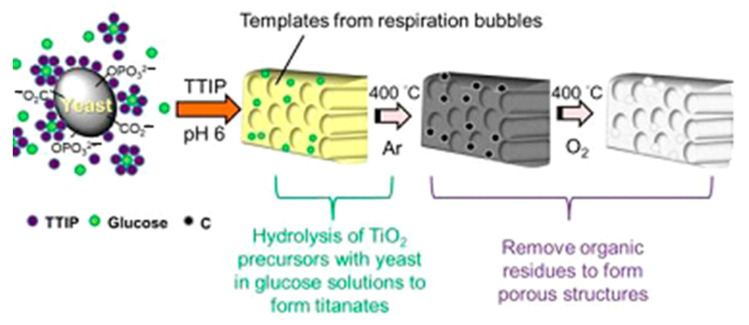
Proposed pathway for the formation of channel-like porous TiO_2_. Reprinted with permission from [31].

The sol-gel process is widely and actively used to obtain nanomaterials based on titanium oxide. Titanium dioxide is considered to be one of the most promising anode materials for lithium-and sodium-ion batteries due to its inherent low toxicity, low cost, and stability [38,116]. Typically, the material needs to be characterized in terms of porosity, discharge capacity, and retention of capacity over a certain number of cycles to be used effectively in batteries. It has been shown that the structure and morphology of nanostructured titanium dioxide have a significant effect on its electrochemical characteristics [38,117]. Thus, careful control of the structure and properties of synthesized titanium dioxide is extremely important for obtaining a new efficient and stable anode material for lithium- or sodium-ion batteries. The use of yeast cells as a biomaterial in the formation of sol-gel matrices based on titanium oxide enables us to obtain a hierarchical porous structure that reproduces the microstructure of yeast cells. Wen et al. used baker’s yeast as a biomaterial with subsequent annealing at 450 °C. The resulting samples had a porous structure consisting of macropores (1.5–2.5 μm) and pore walls containing mesopores (9.78 nm). When tested in sodium-ion battery anodes, porous TiO_2_ showed a discharge capacity of approximately 255.98 mAh/g and a capacity retention of approximately 80% after 100 cycles at 1/3 C. Moreover, the material retained a high discharge capacity of 112.93 and 84.65 mAh/g even at 5 and 10 °C, respectively [38]. Chiu’s group has developed channel-like meso/macroporous TiO_2_ using titanium tetraisopropoxide as a precursor. This material retained a high capacity of 120 mAh/g even after 80 cycles when tested as an anode material for lithium-ion batteries [31] (Figure 15).

TiO_2_ samples without the presence of glucose, yeast, or both were formed for performance comparison. It is assumed that the high performance of the material is provided by its hierarchical porous structure. Thus, the use of a yeast matrix is a promising way to develop anode materials for rechargeable sodium and lithium batteries.

### 5.4. As Templates

**Figure 16:**
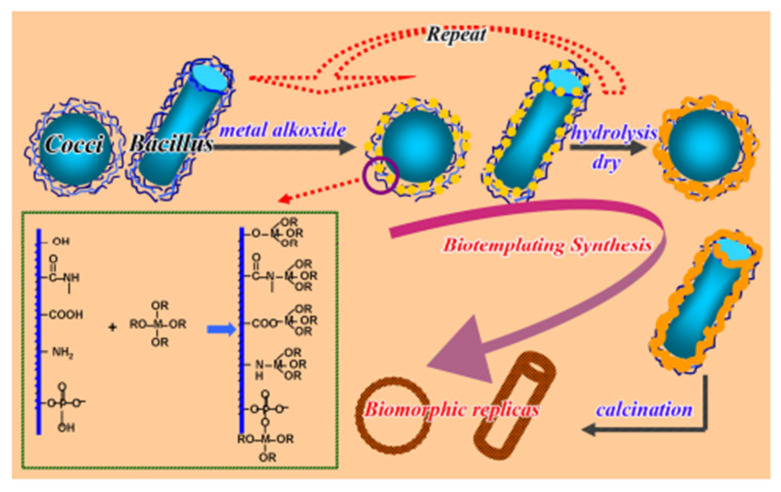
Schematic illustration of the biotemplating synthesis of biomorphic hollow structures via the surface sol-gel process. Reprinted with permission from [54].

Currently, the method of obtaining “molecular imprints” based on the polymerization of functional monomers in the presence of specially introduced target molecule templates (molecular imprinting) is a well-established method for creating nanomaterials with controlled porosity [118]. In this technique, a matrix is formed around a suitable template molecule. The template is then removed, and microcavities of a certain size remain (Figure 16).

Such advantages as a rich variety of shapes, low cost and availability, ecological compatibility, unique configuration, and high repeatability of morphology ensure a high demand for microorganisms as templates in the molecular imprinting process. However, the difficulty of removing microorganisms and their small surface area is a limiting factor for their use in various applications and hinders scaling [119].

Materials obtained by the sol-gel technique combine two important properties: the ability to form very diverse nanomaterials and variable controlled porosity of the matrix. In addition, the ease of fabrication, mild reaction conditions, commercial availability of a wide range of functional monomers, physical rigidity of the resulting matrix, chemical inertness, and resistance to thermal stress and solvent exposure make the sol-gel method using templates attractive for creating cavities in nanomaterials [120].

The combination of sol-gel technology and microorganisms with their subsequent annealing opens up prospects for the development of new materials with controlled porosity and a large specific area. Highly porous materials based on titanium dioxide with bacteria as templates are being developed [38,54,121]. The obtained materials are used in various fields, for example, in the adsorption of gases [54] or in the formation of an anode material in rechargeable sodium batteries [38,121]. Guo and coworkers have shown that hydrogen release from hollow TiO_2_ micro/nanostructures is 3.6 times higher than that of their solid counterparts [54]. The porous structure of the new anode material provided the accessibility of the electrode interface for the electrolyte, reducing the path length for ion diffusion and compensating for volume changes during the cycle [38].

### 5.5. Application of Sol-Gel Hybrid Materials in Catalysis

Sol-gel catalysis is a mature chemical technology that offers unique advantages, including ease of production of materials in various forms (powder, monolith, thin film, coating, etc.) and ease of use. Alkoxides are soluble in organic solvents and readily hydrolysable, making them a convenient source of “inorganic” monomers. The latter subsequently condense into polymer particles. By chemically controlling the mechanisms and kinetics of these reactions (catalytic and reaction conditions), the textural and surface structural properties of the gel can be adapted [122].

The use of sol-gel materials as catalysts has three main advantages. First, the sol-gel matrix (most often silicon dioxide) physically and chemically stabilizes the dopant. This is important in catalytic applications where long-term catalyst stability is required. Second, such materials change the selectivity of the catalyst by determining the approach of the incoming reagents to the active site. Third, they increase the reactivity due to excessive dispersion of the dopant in the ceramic matrix [20]. This is indicated by the use of tetrapropylammonium perruthenate and (2,2,6,6-tetramethylpiperidin-1-yl)oxyl encapsulated in a sol-gel shell. The 75% methyl-modified catalyst used in the oxidative dehydrogenation of benzyl alcohol in supercritical CO_2_ has one of the highest turnover rates for ruthenium-based aerobic catalysts [123]. This material can be reused in subsequent reactions. At the same time, the tetrapropylammonium perruthenate catalyst cannot be recycled in an organic solvent due to the formation of a precipitate of ruthenium particles. This problem is prevented by encapsulating the catalyst in organosilicon shells [124].

Sol-gel encapsulation of the nitroxyl radical markedly improves its chemical stability compared to (2,2,6,6-tetramethylpiperidin-1-yl)oxyl attached to the outer surface of the silica. The destruction of the supported catalyst occurs due to intermolecular quenching of radicals remaining unprotected on the surface of the material. Intermolecular quenching does not occur inside the sol-gel frameworks, which leads to high chemical and physical stability and reactivity [123].

The sol-gel process allows the production of high-performance heterogeneous catalysts. This opens the way for the efficient heterogenization of many homogeneous catalytic systems, which until now could not be commercialized due to the difficulties associated with separating the products from the catalyst.

Thus, ecology, medicine, electrical engineering, template synthesis, and catalysis are the main areas of application of hybrid materials based on living cells of microorganisms immobilized by the sol-gel method. The range of application of hybrid sol-gel materials is due to the presence of a number of unique properties, such as a large surface area, high porosity, biocompatibility, good mechanical strength, and ease of preparation.

## 6. Use of Organic Matter to Improve Microbial Viability

Currently, there are no specific studies on the interaction between the sol-gel matrix and living encapsulated cells. A number of research articles describe the harmful effect of alcohols, which form in hydrolysis reactions and remain in the reaction solution, which is a problem for the long-term viability of encapsulated cells [125].

To create more stable structures, various additives have been used that allow obtaining a biocompatible microenvironment to preserve the long-term viability of captured cells. These additives can be polymers, for example, alginates, carrageenans, agar, guar gum, cellulose, pectin, and chitosan, including their derivatives and polypeptides [41].

Additives can improve biocompatibility, for example, by improving surface interactions between the gel matrix and the biological component [126]. Polyethylene glycol acts as a surfactant that reduces the interfacial energy between liquid and gel, which leads to an increase in porosity due to improved interaction with encapsulated biological components [127].

Gel formation of polymers such as PVA via multiple freeze-thaw steps is widely used to solve this problem. This process promotes the formation of hydrogen bonds and the production of stronger hydrogels. However, polymerization at temperatures below 0 °C adversely affects the immobilized cells, leading to the loss of their functionality and metabolic activity. Therefore, various methods of gelation of PVA have been developed, for example, the formation of a polymer using boric acid. However, the resulting matrices were fragile and unstable. Thus, the most appropriate and efficient process at room temperature without the use of any costly and harmful chemicals is LentiKats technology [128]. A technique that could be an extension or alternative to the conventional alkoxide sol-gel process is the freeze-gel technique for biocomponent/cell immobilization. This method can be used to fabricate biological ceramic composites. This material is an inexpensive, porous composite without cracks and with almost zero shrinkage due to the use of colloidal SiO_2_ and a biocomponent in an aqueous medium. This customizable method allows linking the freezing step that was required for the sol-gel transition to the retention of immobilized bacteria and their possible division within biospheric cells. Freeze-gelation was used to analyze immobilized *Bacillus sphaericus* for cell viability, storage capacity, and metabolic activity. The resulting biocomposites have the potential to increase mechanical stability and maintain the viability of immobilized microorganisms for several months [41]. An optical biosensor for the determination of methyl parathion was obtained by immobilizing *Sphingomonas* sp. into polyethyleneimine-functionalized silica. At the same time, the stability of the biohybrid increased 10 times, from 18 to 180 days during storage. In addition, the sensitivity and stability of the biosensor itself have increased [56]. Controlled evaporation of alcohol, which is a co-product of hydrolysis and condensation of alkoxide precursors, in vacuum allows up to 95% of viable cells to be preserved [52]. In addition, the use of organically modified TEOS and glycidoxypropyltrimethoxysilane in the presence of PEG rather than pure silicagels as the host matrix showed a marked improvement in the viability of encapsulated cells (18 vs. 6 days, respectively) [52]. However, in a study [129], the captured microalga *Chlamydomonas reinhardtii* showed a slower growth rate than free cells and did not reach the stationary phase when immobilized in silica hydrogels. The reason for this difference may be the diffusion limitation shown by the biomass gradient. This means that the cells at the top of the gel were adequately supplied with nutrients, whereas the cells in the lowest third of the gel were nutrient depleted.

**Figure 17:**
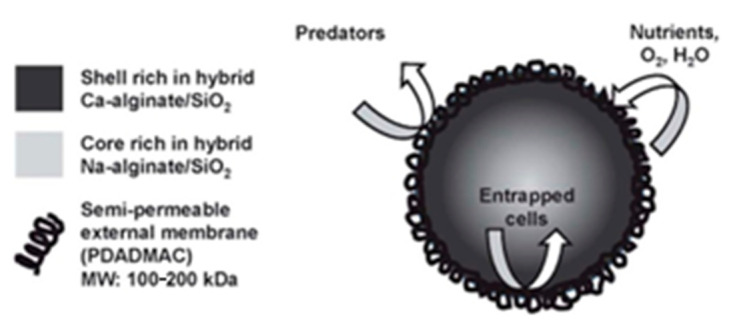
Schematic illustration of the biotemplating synthesis of biomorphic hollow structures via the surface sol-gel process. Reprinted with permission from [130].

Alginate capsules are used to protect microorganisms from harmful environmental factors. This contributes to the increase in the stability of the biomaterial and in the efficiency of functioning of cells. One of the most commonly used encapsulation methods is the immobilization of biocatalysts in silica-coated alginate beads. The encapsulation of *Dunaliella tertiolecta* in alginate/SiO_2_ hybrid matrices produces an optically transparent and strong material with significant porosity without loss of microorganism catalytic activity (Figure 17) [130].

**Figure 18:**
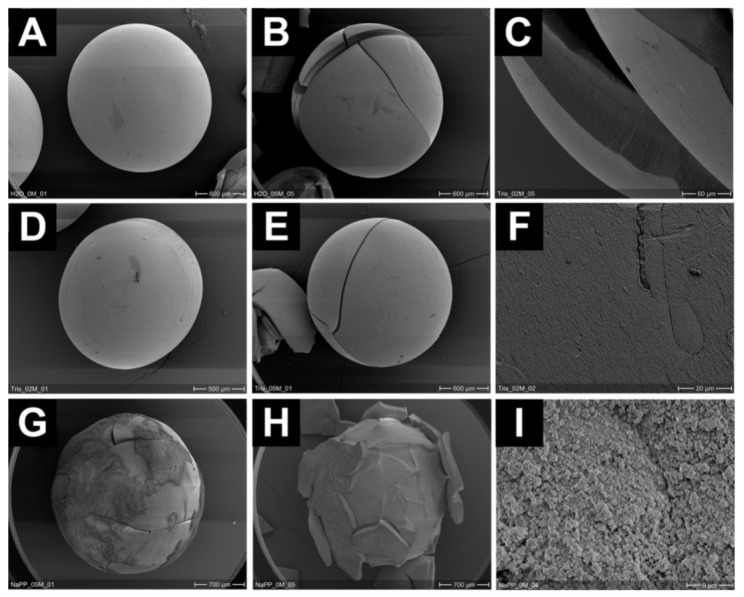
SEM images of silica-coated alginate beads before and after mechanical stress test. Alginate solution was prepared in water (**A**,**B**), Tris/HCl buffer (**C**–**F**), or sodium phosphate buffer (**G**–**I**), and beads were cured in 0.5 M calcium chloride. Coating was performed with pure TMOS for all beads shown. (**A**,**D**,**G**): Beads before the stability test. (**B**,**E**,**H**): Cracked beads after the stability test. (**C**): Close-up of breaking edge. Surface details of a bead prepared from Tris−HCl-alginate (**F**) and sodium phosphate-alginate (**I**). Reprinted with permission from [131].

Rehbeins’ group obtained alginate beads coated with silica shells of varying chemical compositions. The authors studied the resistance of these beads against mechanical stress (Figure 18). It was found that the structural integrity of coated beads is highly dependent not only on the composition of the coating material but also on the method of preparation of the alginate core [131].

Therefore, the use of organic substances to increase the viability of microorganisms immobilized by the sol-gel method is an interesting approach that has been sufficiently developed in the literature. The freezing-gelation approach can be used most effectively to solve the problem of inactivation of living microorganisms. Thus, the obtained hybrid materials will retain the viability of microorganisms for up to several months.

Further summary will be provided in Table 1 and discussed below.

## 7. Conclusions

We have provided a detailed comparative analysis, which helps finding a suitable material, estimate the properties, and assess the best combination of precursors, organic components, and biomaterials (Table 1). The simplicity of the synthetic procedure on the one hand and the variety of morphologies and applications of the obtained nanomaterials on the other hand make the considered systems highly advantageous (Table 1). The following main points can be briefly summarized.

Both bacteria and yeast cells are used in the formation of hybrid biomaterials. However, the latter are used more often, as the alcohol released during the sol-gel process is not as harmful to yeast as it is to bacteria. At the same time, the utilization of *Escherichia coli*, *Pseudomonas*, *Streptococcus*, and *Bacillus* bacteria makes it possible not only to immobilize the biomaterial but also to study its properties and structure.

Most often, organosilicon substances act as precursors when creating a matrix for biomaterial immobilization. Titanium-containing precursors are used less frequently. However, in the last few years, research on the use of precursors containing aluminum and cerium has been intensively developed.

Nanomaterials such as bioceramics or thin films can be obtained by the sol-gel method when creating hybrid biomaterials. It depends on the type of precursors and microorganisms used and the scope of the resulting nanomaterial.

Biohybrids obtained by the sol-gel technique are often used in ecology, medicine, in the creation of batteries, and as templates. The resulting biohybrids can be used in ecology because the synthesized material protects immobilized microorganisms from harmful factors. At the same time, the porous sol-gel matrix does not prevent the penetration of nutrients into the cell and the leaching of waste products. In addition, many microorganisms have a wide range of enzyme systems and are able to oxidize a broad spectrum of substances, which is important in assessing the integral characteristics. The porous structure and large surface area of sol-gel materials based on silicon dioxide or titanium ensure their efficient use in medicine. In this case, microorganisms can act as templates, and the resulting mesoporous materials can be used to load pharmaceuticals, for example.

We believe that this field will undergo rapid growth in the coming years, and the application areas will widely expand to include more chemical fields, for example, catalysis, synthesis, and medicine. Many new results and uses can be anticipated soon.

## Figures and Tables

**Table 1:** Composition, properties, and applications of biohybrid materials obtained by the sol-gel method.

Precursors	Organic Component	Biomaterial	Properties and Applications	Reference
SiO_2_	chitosan derivative (CHT) containing completely natural quaternary amine fragments	human mesenchymal stem cells (hASC)	To get new generations of hybrid materials with silica shell functionalization by modifying the cell surface. These materials can be applied in various fields such as tissue engineering, biosensors, drug delivery, and targeted cell therapy.	[132]
TEOS, MTES, phenyltriethoxysilane, TMOS, aminopropyltriethoxysilane, colloidal silica, sodium silicate	polyethylene glycol, glycerin, chitosan, trehalose, N-(3-triethoxysilylpropyl)gluconamide (GLTES)	*Escherichia coli*	To use bacteria for the atrazine utilization and biosorption of Cd^2+^ ions. Efficiency is due to the presence of a hydrophobic additive in the required ratio. Search for such a ratio for the most efficient utilization of atrazine. To study the processes of encapsulated bacteria division and the preservation of their biological activity. To study the structure of the obtained material in the presence of various organic components to increase the long-term operation of the biomaterial, as well as for research the activity of microorganisms immobilized in the sol-gel matrix during the aging of the immobilizing material.	[13,19,27,32,48,49,50,51,52,99]
TEOS, GLYEO (3-glycidyloxypropyl)-triethoxysilane		*Pseudomonas fluorescens, Rhodococcus ruber, Haematococcus pluvialis, Nannochloropsis limnetica, Botryococcus braunii, Chlorella vulgaris*	To obtain thin films. The catalytic activity of microorganisms was studied by the intensity of glucose oxidation.	[29]
TEOS, titanium isopropoxide, triethoxysilane (TREOS), diethoxydimethylsilane (DEDMS), diethoxymethylsilane (DEMS)	glycerol	*Saccharomyces cerevisiae*	To study the long-term stability of immobilized material. To assess the rate of gene expression. For the development of biosensors. To study the morphology of the resulting structures and the thickness of the resulting films.	[34,50,51,63,64]
Aluminosilicate		*Rhodococcus ruber*	To study the biological activity, mechanical strength, and structure of biologically active ceramic composites derived from *Rhodococcus ruber* bacteria and capable of degrading phenol. The immobilized cells showed no decrease in activity when stored for several months at 4 °C. They can be stored for up to 12 months without losing their biological activity.	[21]
Silicon oxide		*Mesotaenium* sp. *Synechococcus* sp.	To produce optical biosensor for detection of heavy metal ions Cd^2+^, Cr^6+^ and Zn^2 +^ in aqueous media.	[97]
Colloidal SiO_2_ SiNa/LUDOX 1/1		Genus *Bacillus*	To maintain the viability of immobilized microorganisms for several months. To improve mechanical stability. For the determination of heavy metal ions.	[41,47]
TEOS, MTMS, TMOS, sodium silicate	glycerol	*Pseudomonas* sp.	The cells retain high viability for 365 days after immobilization when stored in phosphate buffer at 4 °C. Immobilized cells are able to efficiently produce biosurfactants and can participate in the biodegradation of azo dyes.	[43,44,45,93]
Colloidal silicon dioxide		*Streptococcus lactise*	To increase the catalytic activity of immobilized cells and the protective function of the matrix.	[46]
Sodium silicate		nodule bacteria of the genus *Rhyzobium*	Immobilization of microorganisms in a sol-gel matrix can be considered as an alternative for long-term storage of nodule bacteria.	[53]
TEOS	glycerol	cyanobacteria	To obtain a porous organosilicon capsule. This capsule protects each cell of cyanobacteria from mechanical damage but does not prevent the rapid diffusion of low molecular weight substances through the pores of the capsule.	[26]
Sodium silicate MTMS		*Metylomonas* sp. GYJ3	The activity of encapsulated microorganisms was maintained at 4 °C for 45 days.	[58]
TMOS		*Paracoccus denitrificans*	To determine the content of phospholipids of fatty acids using an optical sensor.	[27]
TEOS		*Yarrowia lipolytica*	The resulting biohybrid material has the ability to remove Cr (III) and Cr (VI) pollutants with high efficiency and without special pre-treatment from water.	[65]
	Polyvinyl alcohol and 4-vinylpyridine	*Trichosporon cutaneum*	To maintain the viability of encapsulated cells. Arthroconidia are formed in extracellular material and play an important role in maintaining the long-term viability of microorganisms. This may be due to the fact that arthroconidia have the ability to withstand environmental stresses. A biosensor based on encapsulated yeast has been used to analyze biochemical oxygen demand in contaminated wastewater.	[66]
TEOS		*Paracoccus denitrificans*	Monitoring the state of cells (live/dead) immobilized in silica gel by determining phospholipid fatty acids.	[69]
TEOS		*E. coli,* *Staphylococcus aureus*	SiO_2_ nanoparticles are biologically inert and have an antimicrobial effect against *E. coli* and *Staphylococcus aureus* bacteria. Nanoparticles are non-toxic, which was shown in a study on a human lung epithelial cell line (A549)	[63]
TEOS, aluminum silicate	glycerin, trehalose	*Aquincolatertiaricarbonis* L108	Development of biofilters that are able to decompose difficult-to-oxidize methyl tret-butyl ether and ethyl tret-butyl ether. Immobilized biomaterial can be stored up to 8 months.	[71]
TEOS		*Lodderomyceselongisporus*, *Candida norvegica*, *Debaryomyces fabryi*, *Pichia carsonii*	Development biocatalysts of the next generation. They provide longer catalytic activity of immobilized cells.	[67]
TEOS		*Humicola lutea, Bacillus* sp.		[72]
TEOS, tetra(n-propylamino)silane		*Chlamydomonas reinhardtii ent*	Comparison of cell viability immobilized with silane precursors and immobilized with sodium silicate.	[82]
Tetraethyl orthosilicate		*Trametes versicolor*	The characterization of the free silica and *Trametes versicolor* cells in ceramic matrices was carried out by using scanning electron microscope, transmission electron microscope, Fourier transform infrared spectrophotometer, nitrogen adsorption–desorption measurement, and catalytic activity assay.	[77]
TEOS		*Algae*	Biosorption of heavy metals	[78]
TEOS		*Ralstonia eutropha* MTCC 2487	Immobilized bacteria utilize arsenic As (V)	[90]
TEOS, GLYEO (3-glycidyloxypropyl), TEOS		Microalgae cells *Haematococcus pluvialis*	Microalgae immobilized in sol-gel layers can be used for the biotechnological production of astaxanthin. It has been shown that the formation of astaxanthin during cultivation can be increased by the combined use of Fe^2+^ compounds with NaCl or hydrogen peroxide as stress factors.	[68]
Sodium silicate		*Synechocystis* sp. PCC 6803	To study gene expression of encapsulated microorganisms.	[91]
diamino-functional silane N-(2-aminoethyl)-3-aminopropyltrimethoxysilan, TEOS	Sodium alginate	*Chlorella vulgaris*	To improve the stability of *Chlorella vulgaris* cells in saline solutions, as well as to achieve stable reproducibility of the obtained materials.	[96]
TEOS	chitosan	*Gluconobacter oxydans*	A mediator whole-cell biosensor with acetic acid bacteria was created and characterized. Bacteria were immobilized on a graphite electrode using a hybrid composite obtained by the sol-gel method.	[98]
TEOS, MTES	PEG	*Pichia angusta, Cryptococcus curvatus*	To create a biosensor for the utilization of lower alcohols and determine their concentration.	[101]
3-aminopropyl trimethoxysilane TMOS, DiMe-DMOS, TEOS, silicon oxide	poly(vinylalcohol), 4-vinylpyridine (PVA-g-P(4-VP)) PVA, 4-vinylpyrrolidone	*E. marius*, *B. horikoshii,* *H. Marina* *B. licheniformis,* *D. marisand,* *M. marinus,* *Trichosporon cutaneum*, *Bacillus subtilis*	To create a BOD biosensor.	[66,103,104,105]
Silica nanoparticles	polyethyleneimine (PEI)	*Sphingomonas* sp.	To improve the previously created optical microplate biosensor for methyl parathion based on *Sphingomonas* sp.	[56]
TEOS		*Escherichia coli, Chromobacterium violaceum,* *Lodderomyces elongisporus*	To create biocatalysts capable of joint utilization of organic substances.	[107]
TEOS		*Lodderomyceselongisporus*, *Pichia carsonii*, *Candida norvegica*, *Debaryomyces fabryi*	To create biocatalysts for organic synthesis.	[108]
TEOS		*Citrus aurantium Lextract*	Citrus flavonoids were immobilized in a sol-gel matrix. Sol-gel synthesis and structure formation were investigated using X-ray diffraction patterns (XRD), Fourier transform infrared spectroscopy (FTIR), scanning and transmission electron microscopes (TEM). The resulting nanohybrid materials had an agglomerated amorphous structure with a particle size of 171–199 nm.	[111]
TEOS		Tannins from *Acacia mearnsii*	The best results were obtained using the silicate sol-gel method. Only hybrid materials prepared using the silicate route have demonstrated good antimicrobial activity. The bactericidal activity of the materials was close to that of pure tannins. Thus, the sol-gel process prevents the loss of tannin through oxidation and hydrolysis. The tannin can be released in an aquatic environment in a controlled manner.	[114]
TEOS		*Chlamydomonas reinhardtii*	To increase cell viability. To develop a low ethanol synthesis method.	[129]
Titanium tetraisopropoxide, TiSO_4_		Yeast cells	Cells were used to form a material with a given structure (they were then burned out). The material can be applied as the anode of a lithium-ion battery.	[31,38]
Titanium (IV) oxide (immobilized on silicon oxide or activated carbon support)			Material-catalyst for utilization of organic pollutants.	[35]
Ti(OEt)_4_	triethanolamine	*A. chlorophenolicus,* *P. anomala,* *Lb. plantarum*		[37]
Bis(ammonium lactato) titanium dihydroxide (IV)	Poly (diallyldimethylammonium)chloride, alginate		To create a mesoporous and biocompatible material as a repository of animal cells for use in cell therapy.	[85]
Butoxide tetraethyl titanium		Yeast	Catalytic tests have shown that the new N-TiO_2_/MnO_2_ hollow nanosphere has a higher photodegradation activity against formaldehyde gas under visible irradiation than commercial TiO_2_. This is explained by the higher surface area (160 m^2^g^−1^) of the hollow structure. The catalytic efficiency of the developed material was more than 90%, which is about 10 times higher than that of the traditional TiO_2_-P25 catalyst.	[112]
Aluminum chloride (thermohydrolysis in alkaline medium)	glycerol	*Escherichia coli*	To use alumina, the rate of formation of the material is higher, and the survival of microorganisms is lower compared to the material obtained on the basis of silicon oxide precursors.	[36]
Aluminosilicate		*Rhodococcus ruber*	To study the biological activity, mechanical strength, and structure of biologically active ceramic composites obtained on the basis of *Rhodococcus ruber bacteria* capable of degrading phenol. The immobilized cells showed no decrease in activity when stored for several months at 4 °C. They can be stored for up to 12 months without loss of their biological activity.	[21]
		Incubated wet yeast	To create a BOD-biosensor	[86]
Ce(NO_3_)_3_		*Morinda citrifolia*	IR spectroscopy has proven the production of cerium oxide nanoparticles, which are formed due to the extract of *Morinda citrifolia*. The TEM method demonstrated the formation of spherical nanoparticles.	[33]
CeO_2_ nanoparticles (embedded in transparent silica hydrogel, TEOS)		*Chlorella vulgaris*	The resulting materials have protective properties due to the applied precursors. The immobilized cells were protected from UV, H_2_O_2._	[39]
